# Supplementary material for: 2D and 3D Triangulation Are Suitable In Situ Measurement Tools for High-Power Large Spot Laser Penetration Processes to Visualize Depressions and Protrusions before Perforating
Source: Materials (Basel). 2022 May 24;15(11):3743. doi: 10.3390/ma15113743 (PMC9181421; doi:10.3390/ma15113743)
Supplement: Supplementary file 1 [file materials-15-03743-s001.zip › materials-1703215-supplementary.pdf]

Supplementary Materials: 2D and 3D Triangulation Are Suitable In Situ Measurement Tools for High-Power Large Spot Laser Penetration Processes to Visualize Depressions and Protrusions before Perforating

Stefan Reich *, Alexander Göbel, Marcel Goesmann, Dominic Heunoske, Sebastian Schäffer, Martin Lueck, Matthias Wickert and Jens Osterholz

Caption for video SI_Crosssection.avi:
Temporal profile change of three laser penetration processes with spot diameters $D4\sigma =$ 16 mm, 22 mm and 31 mm from left to right, respectively. Besides the temporal scaling, the melt pool dynamics for the two larger spot sizes is comparable. The molten area enlarges mainly around the laser impact point. With the small spot size, however, the melt pool enlarges mainly upwards. For more details, see main text.

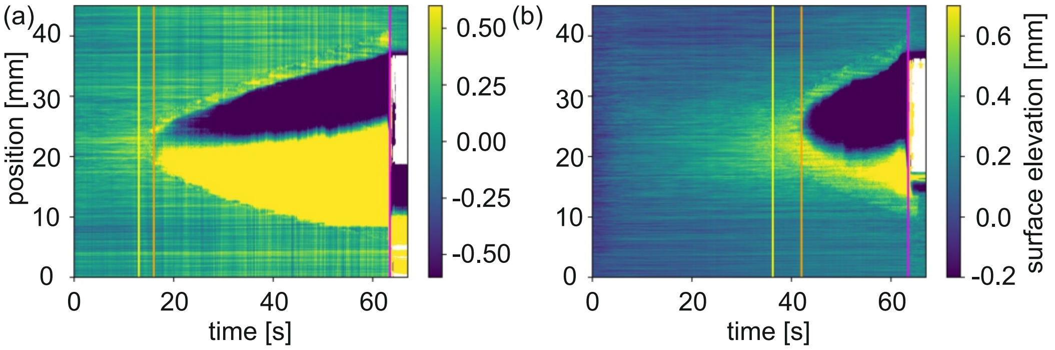

Figure S1. Temporal change of the surface on the front (a) and back side (b) with a color range highlighting small changes compared to the origin. Around the area of strong surface changes (protrusion and indentation), an area of constant small changes is observed. This area starts earlier (yellow lines) than the surface melt (orange line) and develops further outside the melted area.
